# Microscopic and ultrastructural evidences in human skin following calcium hydroxylapatite filler treatment

**DOI:** 10.1007/s00403-017-1734-3

**Published:** 2017-03-21

**Authors:** Nicola Zerbinati, Edoardo D’Este, Pier Camillo Parodi, Alberto Calligaro

**Affiliations:** 1 Department of Surgical and Morphological Sciences, University of Insubria, Varese, Italy; 2 Dermatology Department Centro Medico Polispecialistico, Pavia, Italy; 3 Department of Medical, Experimental and Clinical Sciences, University of Udine, Udine, Italy; 4 Department of Public Health, Experimental and Forensic Medicine, Histology and Embryology Unit, University of Pavia, Pavia, Italy

**Keywords:** Skin, Hydroxylapatite, Ultrastructure

## Abstract

This study uses light and electron microscopes to gain a better knowledge of the interactions of calcium hydroxylapatite filler with the connective tissue of the skin and the modifications of the human deep dermis, after 2 months of treatment. Some morphological evidences of this observational study of filler treated tissue support-specific mechanism involved in the structural modifications of both filler microspherules and cells of the connective tissue. They demonstrate the absence of any immunological reaction and show that the used filler is modified very slowly over time by the action of cells of the connective tissue closely related to the filler without any activity of phagocytosis. Furthermore, associated with the modifications of the filler, evidences of stimulatory effects on dermal fibroblasts are reported.

## Introduction

Recently, many patients require extensive aesthetic treatments and some of them choose conventional surgery as treatment of first choice. Injectable soft tissue fillers are extensively used for many age-related skin signs such as the rejuvenation of limited areas of the face, the neck and other regions. Many materials have been used as fillers, and many procedures have been employed in the last 15 years for the rejuvenation of some regions of the body. A lot of controversial data are available in scientific literature, but in the last couple of years, a clear evidence has emerged on the safety and tolerability of calcium hydroxylapatite filler, which has been used for over 10 years, as an effective and safe treatment option for a variety of aesthetic indications [1–3]. This study reports some observations, 2 months following the injection of calcium hydroxylapatite (CaHa) filler on abdominal skin of patients who requested for the removal of redundant skin. The aim of this study is to deepen the knowledge of the modifications of the injected material and its interactions with the extracellular matrix of the connective tissue and related cells. It also aims to evaluate at cytological level the possible stimulatory remodelling effects.

## Materials and methods

Five patients (female) scheduled for redundant abdominal skin removal were enrolled for this study. Two months before surgery of each subject, a local subdermal anaesthesia with 1 ml lidocaine 1% solution was administered using a 30-gauge syringe. A small volume (0.3 ml) of calcium hydroxylapatite filler Radiesse^®^ (Merz, Frankfurt, Germany) was also injected with a 25-gauge 1-inch-long needle in the subcutaneous plane during a slow withdrawal of the syringe. A superficial (epidermal) mark was made on the injection site of the abdominal skin. Two months later, the surgical excision of the redundant skin permitted the collection of two kinds of specimens, one from the landmarked area and another from a non-treated zone as control. Both were processed using light and electron microscopy. Observations were performed on each sample to detect any microscopic and ultrastructural changes.

### Light microscopy

Skin samples were immersed in a 4% paraformaldehyde/phosphate buffer solution for 24 h, after which they were processed for light microscopy through dehydration, embedding in paraffin and sectioning. Some sections were stained with haematoxylin and eosin (Merck, Darmstadt, Germany) for a general overview of samples. Semithin sections (0.2 µm) from epoxy resin-embedded samples (see “Electron microscopy” section) were stained with toluidine-blue (Merck, Darmstadt, Germany) and observed with a Zeiss Axioplan microscope (Carl Zeiss, Oberkochen, Germany) provided with specific filters for differential interference contrast. Image recordings were obtained by a high definition 5.24 megapixel CCD (charge-coupled device) color camera head Nikon DS-Fi2 (Nikon, Tokyo, Japan) coupled with the microscope.

### Electron microscopy

Bioptic samples were immersed in a 2.5% glutaraldehyde [EM grade (Polyscience, Eppelheim, Germany)] —4% paraformaldehyde in 0.1 M sodium cacodylate buffer solution (pH 7.3) for 6 h at 4 °C. Samples were post-fixed for 2 h in osmium tetroxide (Sigma-Aldrich, St. Louis, MO, USA) 1.33% in 0.1 M s-collidine (Merck, Darmstadt, Germany) buffer and dehydrated in a graded series of ethanol (30, 50, 70, 90, 100%). Finally, specimens were embedded in epoxy resin [Epon 812 (Sigma-Aldrich/Fluka, St. Louis, MO, USA]. Semithin (0.2 µm) and ultrathin (40–60 nm) sections were obtained using the ultramicrotome Reichert Ultracut S (Leica Microsystems, Wetzlar, Germany) provided with a diamond knife. Ultrathin sections, after the collection on 200 mesh grids, were counterstained with lead citrate and uranyl acetate (Merck, Darmstadt, Germany). Observations and electron micrographs were made at a transmission electron microscope Zeiss EM 10 (Carl Zeiss, Oberkochen, Germany) operating at 80 kV with an objective aperture of 30/60 µm. Images were recorded on Kodak 4489 Electron Image film (Kodak, Rochester, USA) and finally digitized with an Epson Perfection V750 Pro scanner Seiko Epson Corporation, Suwa, Nagano, Japan) at 1200 dpi.

## Results

### Light microscopy

**Fig. 1:**
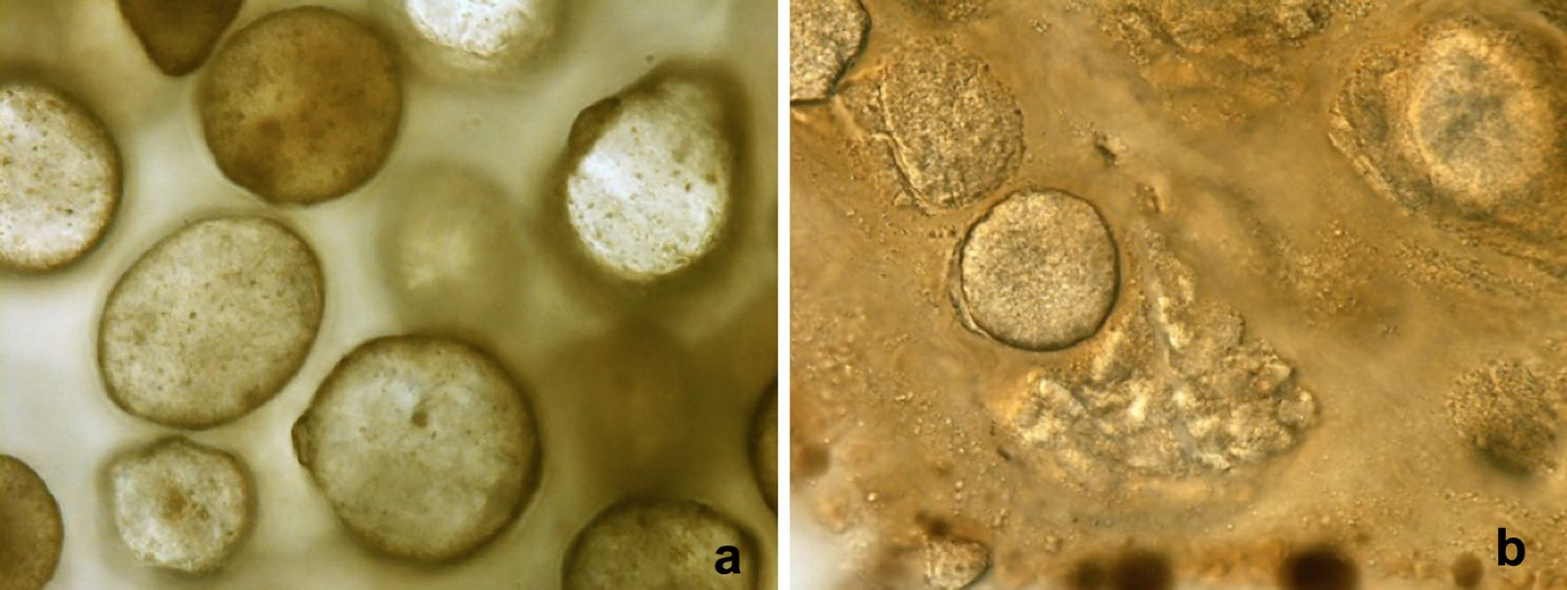
Light microscopy. **a** High magnification of microspherules suspended in salt buffered solution of pH 7.35. Unstained preparation. **b** Rough thick section of deep dermis, from epoxy resin embedding for electron microscopy, observed at the light microscope. Microspherules show different features, suggesting specific interactions with the components of the surrounding connective tissue

Before injection, calcium hydroxylapatite filler appeared to be constituted by spherical structures (microspherules) sized 25–45 µm. In the filler preparation (Fig. 1a), microspherules showed well-defined and smooth surfaces, with a content of microgranules randomly dispersed in an optically poor dense matrix. Some rough thick sections of deep dermis, after fixation of biopsies with paraformaldehyde/glutaraldehyde and osmium tetroxide processed for electron microscopy, were observed under the light microscope. In these sections, some microspherules appeared homogeneous, while others were showing inhomogeneous contents (Fig. 1b). Others seemed to have lost their identity, with their content appearing disrupted in small blocks of material.

**Fig. 2:**
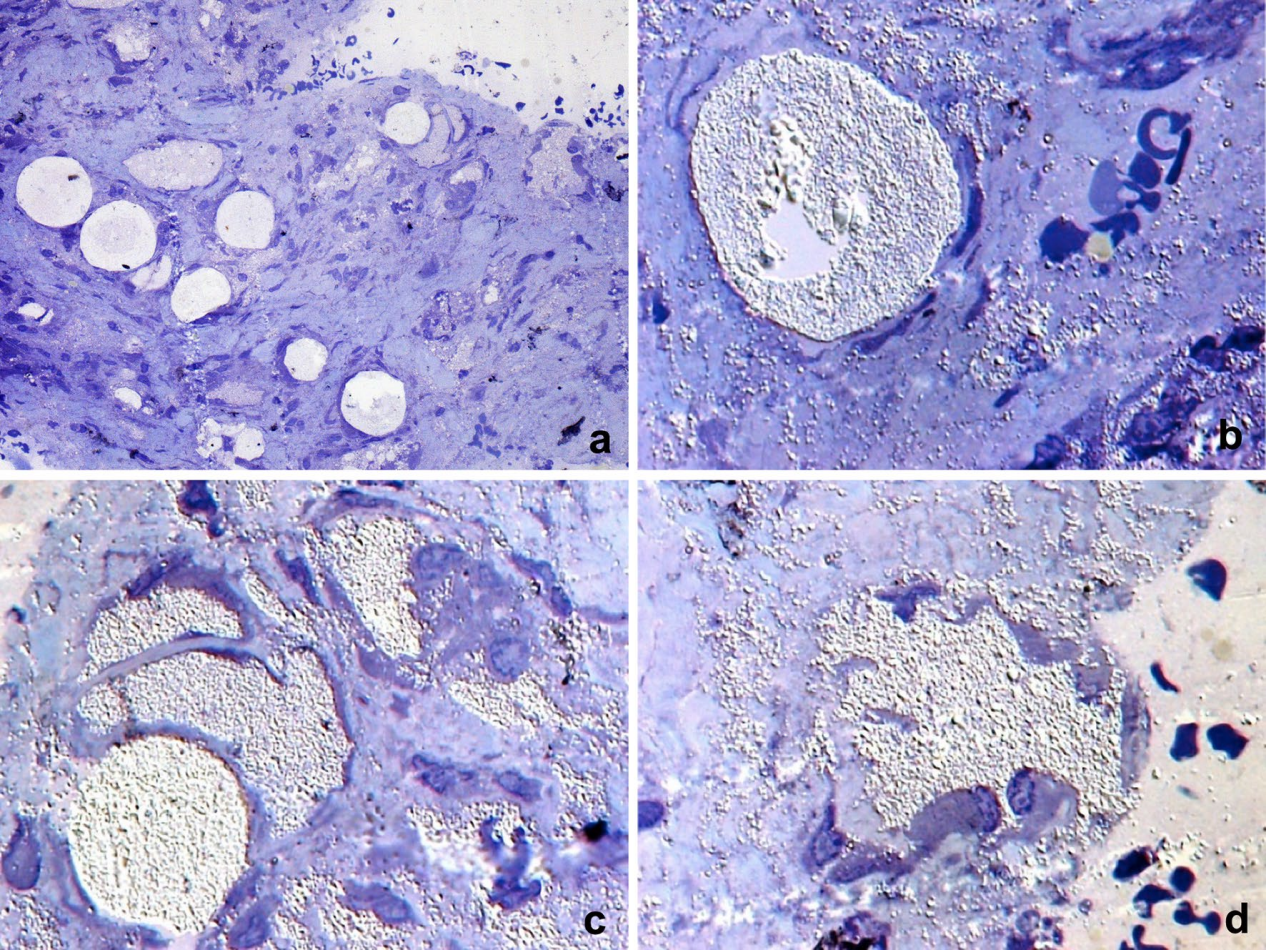
Light microscopy. Semithin sections from epoxy resin embedding stained with toluidine-blue. **a** Low magnification: some microspherules appear rounded, with a clear and highly refractive content, and surrounded by single or multiple flattened cells. Others, differently shaped, show a less refractive content and appear related with many cells all around them. **b** High-resolution interferential contrast of a single microspherule with flattened cells close to its surface. In the surrounding matrix, many microgranules, single or forming aggregates, are observable. **c** High-resolution interferential contrast of microspherules with highly refractive material inside. Variously shaped microspherule residuals closely related with peripherally located flattened cells, and dispersed microgranules in the extracellular matrix. **d** High-resolution interferential contrast of a microspherule in the course of fragmentation. Some cells are located peripherally, and others appear projecting inside the microspherule. No microgranules are detectable inside cells. Numerous microgranules dispersed in the neighbouring areas are also visible

The different features of the microspherules and the close relationships with the surrounding connective tissue suggested specific interactions at the microspherules periphery with the connective tissue cells and matrix components. The observations of semithin sections (0.2 µm) stained with toluidine-blue at low magnification showed some microspherules as round shaped, with a clear and highly refractive content, surrounded by flattened cells. Others were differently shaped, showed a less refractive content and appeared related with many cells around them (Fig. 2a). At higher magnification (Fig. 2b), using a high-resolution and high aperture (NA 1.4) oil-immersion objective at the interferential contrast microscope, a single microspherule with flattened cells with expanded cytoplasm close to its surface was observable, without any apparent specific relationship or contact between them. In the surrounding matrix, many dispersed microgranules, single or forming aggregates, were also observable. The diameter of the smallest single microgranule was less than 1 µm.

The material inside the single microspherule (Fig. 2b, high left) appeared microgranular, compacted and highly refractive. The presence all around microspherule of microgranules dispersed in the connective tissue matrix suggested that their possible origin could have been due to a spread of microspherule material and/or delivery of their content (Fig. 2b).

The most part of microspherules appeared surrounded by elongated and flattened cells often directly and extensively contacting their surface. The surfaces of cells contacting the microspherules appeared smooth. No microgranules were observable inside cell cytoplasm. Single sparse microgranules were observable in the extracellular matrix, free or contacting the external surface of cells (Fig. 2c). In the same Fig. 2c, many cells at the periphery of residual, variously shaped microspherules, were identifiable. Some cells appeared located inside fragmented microspherules or between them. Numerous microgranules dispersed in the neighbouring areas were also visible. Many microspherules, which are more or less fragmented, are clearly observable in Fig. 2c, d.

In Fig. 2d, some cells appear distributed in the peripheral part of a microspherule, while others appear inside it. No microgranules were observable inside cell cytoplasm, single or as storage referred to a possible phagocytic activity. Concerning the presence of cells involved in possible adverse immunoreactions related to the filler, none of the five cases examined in this study showed inflammatory infiltrate or granulation tissue.

### Electron microscopy

**Fig. 3:**
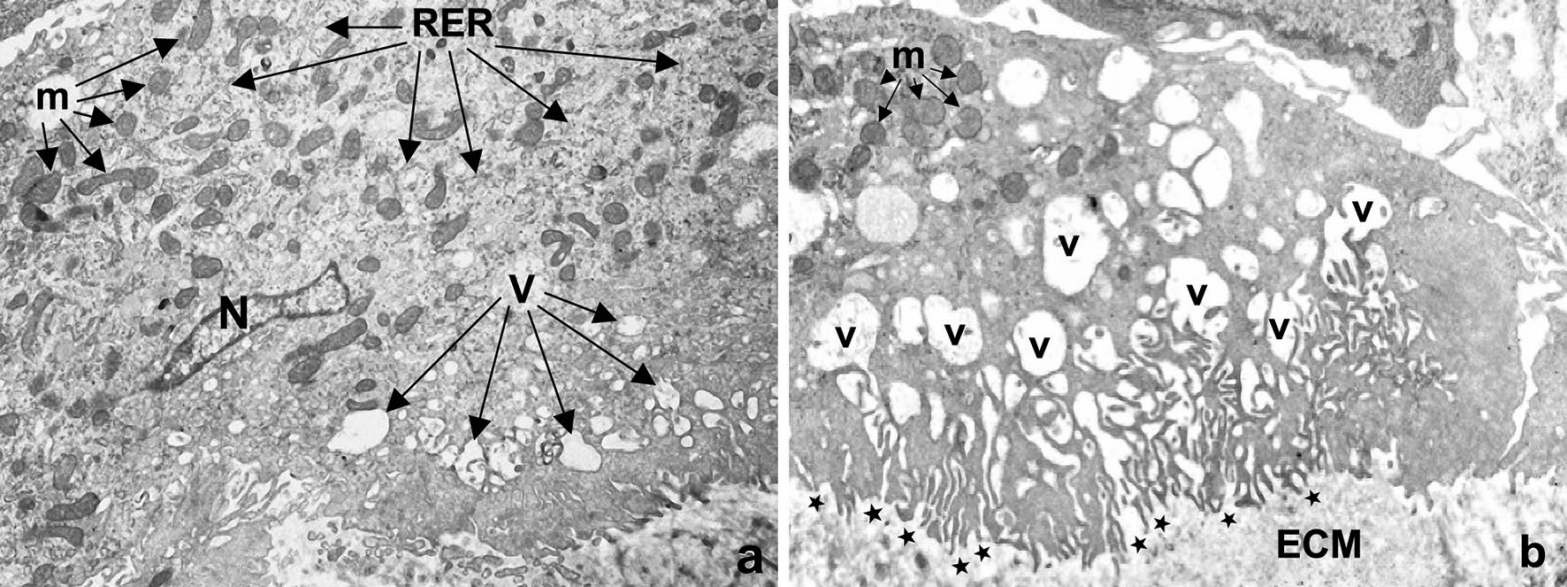
Electron microscopy. **a** The cytoplasm of cells closely related with the microspherules and in the close surroundings of them are rich in mitochondria (*m*), rough endoplasmic reticulum profiles (*RER*) and peripheral variously sized vescicles (*V*). The surface facing microspherule (*low right*) is characterized by numerous membrane invaginations forming a sort of ruffled border. *N* nucleus. **b** The plasma membrane of cells facing microspherules is characterized by numerous deep foldings opening to variously sized vesicles with clear content (*V*). The inside of the vesicles is communicating with the intermicrovillous spaces and their openings (*stars*) to the extracellular matrix (ECM) facing microspherules. In these vesicles, no phagocytotic material is observable. *m* mitochondria

At the electron microscope, the big cells surrounding microspherules show an elongated nucleus and a cytoplasm rich in organelles (Fig. 3a), particularly mitochondria and between them an extended rough endoplasmic reticulum. In the most part of peripheral cytoplasm, a lot of vesicles of different size and clear content are also observable. The surface of these cells is often related with the microspherules, and their membrane facing them is characterized by a sort of ruffled border (Fig. 3a, low right). At high magnification, the ruffled border appear to be formed by a labyrinth of extensions/infoldings of the plasma membrane towards the extracellular matrix, closely related intracellularly with the clear vesicles (Fig. 3b). The continuity of membranes clearly visible in the same Fig. 3b demonstrate the openings of the vesicles into the intermicrovillous spaces communicating with the extracellular space .

The content of the vesicles is clear and homogeneous, without any electron dense material inside, differently from phagosomes, or secondary lysosomes characterizing phagocytic cells. Microgranules or their residuals as secondary lysosomes or residual bodies were never observed in these cells in our study. Realistically, the brush border is the site where the cytoplasmic vesicles discharge their content at the cell surface towards the surface of microspherules.

**Fig. 4:**
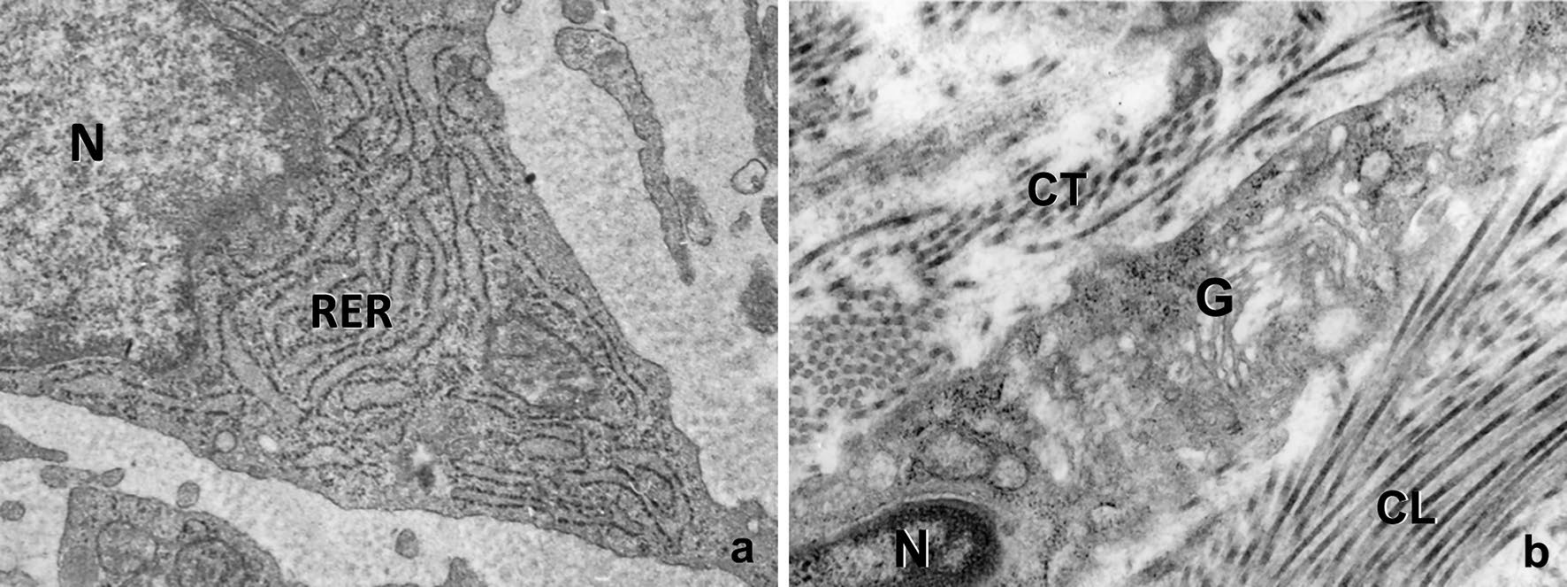
Electron microscopy. **a** The cytoplasm of fibroblasts of the deep dermis appear very rich in cisternae of the rough endoplasmic reticulum (*RER*), often dilated, containing a medium electron-dense and finely filamentous material. The nucleus (*N*) is rich in euchromatin. **b** Into the cytoplasm of fibroblasts, a well represented Golgi apparatus (*G*), formed by stacks of membranes and associated vesicles, is also well detectable. In the extracellular matrix, bundles of collagen microfibrils both longitudinally (*CL*) and transversally (*CT*) sectioned are also observable

In our microscopic preparations two months after CaHA filler injection, fibroblasts of the deep dermis appear highly basophilic under the light microscope. Under the electron microscope (Fig. 4a), the cytoplasm of fibroblasts appeared particularly rich in profiles of the rough endoplasmic reticulum. These profiles are constituted by membranes with attached ribosomes, forming cisternae often dilated, containing a medium electron-dense finely filamentous material. Realistically, this material is representing the molecular precursors of the fibrillar components, mainly collagen, of the extracellular matrix.

A well-developed Golgi apparatus is also visible in fibroblasts (Fig. 4b). This cytoplasmic organelle is the site of both glycosylation of proteins and the synthesis of specific molecular components of the ground interfibrillar substance of the extracellular matrix (proteoglycans, glycosaminoglycans and multiadhesive glycoproteins). In the same Fig. 4b, the extracellular matrix surrounding fibroblast appear rich of bundles of collagen microfibrils, both longitudinally and transversally sectioned.

These specific ultrastructural features observed support a stimulated involvement of fibroblasts in the production of new molecular components of the extracellular matrix, with an active renewal/remodelling of the connective tissue.

**Fig. 5:**
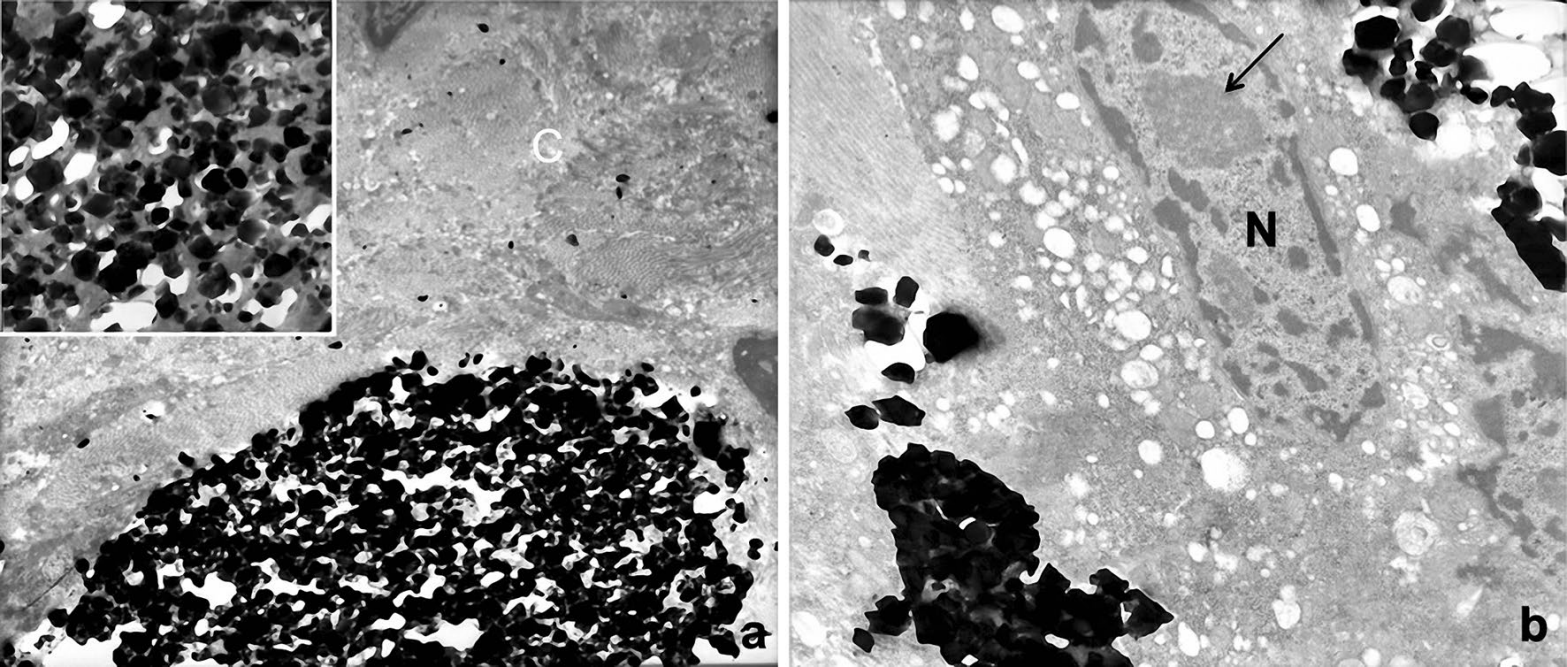
Electron microscopy. **a** Part of a microspherule inside the deep dermis, well identifiable in the half lower part of the electron micrograph, due to the very high electron density of microgranules related to the calcium hydroxylapatite content. The *upper part* of the figure is constituted by compact bundles of collagen fibres (*C*) transversally or obliquely sectioned. Inside them, some microgranules can be recognized due to their high electron density. *Inset* high magnification of a microspherule showing compacted microgranules (*dark*). The *grey material* between microgranules is representing the gel carrier. The *white small areas* associated with microgranules are representing artefacts due to the ripping of the very hard microgranules on the diamond knife making ultrathin sections at the ultramicrotome. **b** Big cell with a nucleus rich of euchromatin (*N*) showing also a well-visible nucleolus (*arrow*), in close relationship with microgranules, free into the extracellular matrix (*left*) and at the surface of a microspherule (*high right* and *low left*). The cytoplasm of the cell is extremely rich of vesicles with a clear and homogeneous content. No intracytoplasmic microgranules or phagocytotic structures are observable

In Fig. 5a, part of a microspherule inside the deep dermis is visible in the lower half of the micrograph. It appeared very dark/black under the electron microscope, due to the very high electron density of the calcium hydroxylapatite content of microgranules. The upper part of the figure is characterized by highly packed bundles of collagen fibres transversally or obliquely sectioned. Some sparse microgranules are detectable between the microfibrils forming the collagen fibres. The inset is a high magnification of the microspherule content (the small white areas associated with microgranules represent artefacts, due to the ripping of the very hard microgranules on the diamond knife during ultrathin sectioning at the ultramicrotome).

In the big cells surrounding the microspherules, some cytological features have to be underlined (Fig. 5b). In the nucleus, the euchromatin (functional chromatin) is clearly predominant, and a big nucleolus is also evident. In the cytoplasm, a lot of vesicles with clear and homogeneous content is visible, both in the most peripheral part of the cell and even in the inner part surrounding the nucleus. In the same Fig. 5b, no secondary lysosomes or phagocytic structures were observable inside the cell. Dark, very electron-dense microgranules, singularly dispersed or forming clusters, were visible extracellularly in the surrounding space.

## Discussion

The calcium hydroxylapatite filler used in this study is a semi-permanent filler constituted by a suspension of 30% calcium hydroxylapatite microspherules (25–45 µm) in a 70% gel containing sodium carboxymethyl cellulose, glycerine and sterile water for injection [4]. It can be stored at room temperature (15–32 °C), and it expires 2 years from the manufacture date (Radiesse^®^ Datasheet). It has been widely demonstrated that it does not stimulate any local or foreign body reaction or adverse systemic effects [5]. Nodule formation following injection of some other CaHa fillers, particularly in the lip, are presenting complications due to wrong injection procedures related to a superficial placement of too large volumes. This is usually as a result of the clinician’s degree of experience with the material [6]. If CaHA filler is injected into the mid or superficial dermis, it will result in visible nodules [7]. The nodules can be removed through puncturing, immediately before the adherence of the microspherules to the adjacent stroma [8]. It can also be removed through the injection of saline and a vigorous massage [9], or with the use of fractional carbon dioxide laser application as demonstrated on the nasojugal fold [10]. In some cases, the nodules require surgical removal [11]. Multiple studies have documented safety, satisfying results, efficacy and longevity in tissue with the use of Radiesse^®^, particularly for the treatment of many areas of the face [1, 12] and through body vectoring technique for tightening the abdomen, thighs and brachial zone [13].

The aim of this study has been to deepen the knowledge of the modifications of the microspherules’ structure and the interactions of microspherules with the extracellular matrix and the surrounding cells and, at the same time, to evaluate the cytological level of a possible stimulatory effect.

In the complex of all the cases observed in this study, following a correct injection procedure of Radiesse^®^, no nodular formation was detected, and microspherules appeared distributed inside the connective tissue of the deep dermis. Some microspherules appeared filled with a fine granular material homogeneously distributed, while others presented a less homogeneous material inside, suggesting specific modifications. Fine microgranular material was also detected, dispersed in the connective tissue matrix’s interstitial space, realistically related to the activity of cells surrounding microspherules. Electron microscopic observations on the interface between microspherules and surrounding cells, as the numerous invaginations of plasma membrane delimitating thin cytoplasmic processes, demonstrated an important increase of the membrane surface. This feature is permitting an extraordinary exchange of molecules through the membranes and between the membranes towards the extracellular matrix. The opening of the numerous cytoplasmic vesicles into the spaces between cytoplasmic processes, communicating with the extracellular space towards the surface of microspherule was suggesting a specific functional interaction. The cytoplasmic vesicles represent realistically primary lysosomes, rich in lytic enzymes synthesized in the rough endoplasmic reticulum and vehicled through cytoplasmic vesicles. Their homogeneous content is reversed extracellularly at the surface of microspherules, thereby determining an enzymatic lysis of the microspherules surface and the gel carrier content.

These features seem very similar to those observed at the surface of osteoclasts facing the bone, where phagocytosis is never detectable It is well known that bone reabsorption is not performed through phagocytosis (osteoclasts do not contain secondary lysosomes), but through an extracellular mechanism switched on by the release of the content of primary lysosomes on the plasma membrane extracellularly [14]. Our observations did never show inside the cells the presence of secondary lysosomes and/or residual bodies, as specific intracellular structures were involved in phagocytosis.

Our morphological findings are supporting indeed an active cellular mechanism with the delivery of hydrolytic enzymes through the clear vescicles opening by exocytosis in the labyrinth of membranes on the cell surface through the ruffled border. In this way, a slow dispersion of the microgranular content in the close extracellular matrix environment, freely into the ground substance and/or between the collagen fibres, would permit a more homogeneous distribution of microgranules. These observations do not agree with some previous research works, reporting lymphocytic infiltrate associated with giant cells engulfing spherules, without migration of the implanted material, and supporting a phagocytotic mechanism [15, 16].

The big cells surrounding the microspherules, realistically originate from monocytes (as histiocytes, and osteoclasts in the bone), which are chemotactically attracted by the microspherules constituting the injected filler. They become fused with one another, and following a specific stimulation by the microspherules, they undergo differentiation. The differentiation of these cells consists of a synthesizing activity in the rough endoplasmic reticulum of specific enzymes, which give origin, through the Golgi apparatus, to a lot of vesicles filled with highly concentrated enzymes which are delivered at the cell surface.

The extracellularly digested material could be considered the cohesive viscous substance linking the hydroxylapatite micrograules (gel carrier). The digestion of such material is responsible for the lack of compactness of microspherules content and of a slow release of the CaHa microgranules into the close extracellular matrix with a diffuse smoothening effect on the skin.

Another feature well demonstrated morphologically in our observations after two months from filler injection is represented by a stimulatory effect on fibroblasts, the constitutive resident cells of the connective tissue responsible for the extracellular matrix components formation and turnover. While some authors were supporting a stimulatory induction to form new collagen as the gel carrier is metabolized [3, 15], others did not give evidence of new collagen deposition, “may be due to the small size of examined samples”, as stated by the authors here cited [16].

In our observations, fibroblasts are particularly rich in rough endoplasmic reticulum profiles often dilated containing a finely filamentous material (Fig. 4a). This material represents the molecular precursors (procollagen) of fibrillar components of the extracellular matrix (mainly collagen) [17]. A Golgi apparatus is also well represented in fibroblasts (Fig. 4b), as the organelle directly involves in the glycosilation of proteins and in the synthesis of the molecular components of the ground interfibrillar substance of the extracellular matrix (proteoglycans, glycosaminoglycans and multiadhesive glycoproteins). The abundance of these organelles (RER and Golgi) in fibroblasts suggests a stimulation of the formation of new extracellular matrix components (both collagen and ground substance). Realistically, it is possible that this stimulation may be due to the cohesive gel carrier associated to calcium hydroxylapatite in the microspherules.

It means, on the basis of our morphological evidences, that the injected calcium hydroxylapatite filler Radiesse^®^ does not only give volume and support to the connective tissue with an aesthetic result, but also has a positive effect of stimulating fibroblasts to produce new matrix, with an effective renewal/remodelling of the skin connective tissue.

## Conclusions

The results of this study 2 months following treatment demonstrate specific modifications of the CaHa filler injected in the deep dermis of the skin. These modifications are due to extracellular interactions of the big cells surrounding the filler microspherules with their constitutive material. Our observations support a mechanism without phagocytosis of the injected material, in other words, without a rapid removal of the injected material by phagocytic cells. Due to the action of the big cells closely related to microspherules, their constitutive material is slowly released filling the extracellular matrix with a long smooth action. The treatment is safe, without stimulation of the immune system and/or any other kind of adverse reactions. Furthermore, our morphological findings demonstrate the stimulation of the fibroblasts activity and a related active regeneration of the connective tissue. This renewal of the molecular components of the extracellular matrix increases support and strength to the skin, constituting an additional, restorative and physiological filling action aesthetically and functionally in sync with the action of the injected filler.

## References

[1] Loghem JV, Yutskovskaya YA, Philip Werschler W (2015). Calcium hydroxylapatite. Over a decade of clinical experience. J Clin Aesth Dermatol.

[2] Pavicic T (2013). Calcium hydroxylapatite filler: an overview of safety and tolerability. J Drug Dermatol.

[3] Yutskovskaya YA, Kogan E, Leshunov E (2014). A randomized, split-face, histomorphologic study comparing a volumetric calcium hydroxylapatite and a hyaluronic acid-based dermal filler. J Drug Dermatol.

[4] Broder KW, Cohen SR (2006). An overview of permanent and semipermanent fillers. Plast Reconstr Surg.

[5] Narins RS, Bowman PH (2005). Injectable skin fillers. Clin Plast Surg.

[6] Tsikas TL (2008). A 52-month summary of results using calcium hydroxylapatite for facial soft tissue augmentation. Dermatol Surg.

[7] Berlin A, Cohen JL, Goldberg DJ (2006). Calcium hydroxylapatite for facial rejuvination. Semin Cutan Med Surg.

[8] Flaharty P (2004). Radiance. Facial Plast Surg.

[9] Voigts R, De Vore DP, Grazer JM (2010). Dispersion of hydroxylapatite accumulations in the skin: animal studies and clinical practices. Dermatol Surg.

[10] Reddy KK, Brauer JA, Anolik R, Bernstein L, Brightman LA, Hale E, Karen J, Weiss E, Geronemus RG (2012). Calcium hydroxylapatite nodule resolution after fractional carbon dioxide therapy. Arch Dermatol.

[11] Jacovella PF (2006). Calcium hydroxylapatite facial filler (Radiesse): indications, technique, and results. Clin Plast Surg.

[12] Berlin AL, Hussain M, Goldberg DJ (2008). Calcium hydroxylapatite filler for facial rejuvenation: a histologic and immunohistochemical analysis. Dermatol Surg.

[13] Cogorno Wasylkowski V (2015). Body vectoring technique with Radiesse for tightening of the abdomen, thighs, and brachial zone. Clin Invest Dermatol.

[14] Teitelbaum SL (2000). Bone resorption by osteoclasts. Science.

[15] Marmur ES, Phelps R, Goldberg DJ (2004). Clinical, histologic and electron microscopic findings after injection of a calcium hydroxylapatite filler. J Cosmet Laser Ther.

[16] Holzapfel AM, Mangat SM, Barron DS (2008). Soft-tissue augmentation with calcium hydroxylapatite—histological analysis. Arch Facial Plast Surg.

[17] Stephens DJ (2012). Cell biology: collagen secretion explained. Nature.

